# A Modular Spectrum Sensing System Based on PSO-SVM

**DOI:** 10.3390/s121115292

**Published:** 2012-11-05

**Authors:** Zhuoran Cai, Honglin Zhao, Zhutian Yang, Yun Mo

**Affiliations:** School of Electronics and Information Technology, Harbin Institute of Technology, Harbin 150001, China; E-Mails: qingdaogancai@sina.com.cn (Z.C.); deanzty@gmail.com (Z.Y.); moyuntc@126.com (Y.M.)

**Keywords:** cognitive radio, spectrum sensing, PSO-SVM, detection threshold

## Abstract

In the cognitive radio system, spectrum sensing for detecting the presence of primary users in a licensed spectrum is a fundamental problem. Energy detection is the most popular spectrum sensing scheme used to differentiate the case where the primary user's signal is present from the case where there is only noise. In fact, the nature of spectrum sensing can be taken as a binary classification problem, and energy detection is a linear classifier. If the signal-to-noise ratio (*SNR*) of the received signal is low, and the number of received signal samples for sensing is small, the binary classification problem is linearly inseparable. In this situation the performance of energy detection will decrease seriously. In this paper, a novel approach for obtaining a nonlinear threshold based on support vector machine with particle swarm optimization (PSO-SVM) to replace the linear threshold used in traditional energy detection is proposed. Simulations demonstrate that the performance of the proposed algorithm is much better than that of traditional energy detection.

## 1. Introduction

Based on the conventional fixed spectrum allocation policy, most available radio spectra have been assigned to registered users, which lead to a serious waste of spectrum utilization. In fact, recent reports from Federal Communications Commission (FCC) have shown that only 30% of the allocated spectrum in US is fully utilized [1]. Cognitive radio, which enables secondary users to utilize the spectrum when primary users are not occupying it, has been proposed as a promising technology to improve spectrum utilization efficiency [2–4], and has three essential components: (1) Spectrum sensing: the secondary users sense the radio spectrum environment within their operating range to detect the frequency bands which are not occupied by primary users; (2) Dynamic spectrum management: cognitive radio networks dynamically select the best available bands for communication; (3) Adaptive communications: a cognitive radio device can configure its transmission parameters (e.g., carrier frequency, transmission power) to opportunistically make best use of the ever changing available spectrum [5].

Spectrum sensing is a fundamental task for cognitive radio. However, there are several factors that make spectrum sensing practically challenging (e.g., low signal-to-noise ratio (*SNR*) of primary users, noise uncertainty, multipath fading). Several sensing methods have been proposed, including likelihood ratio test (LRT) [6–8], energy detection method [9,10], match filtering (MF) method [11], cyclostationary detection method [12,13] and the statistical covariances-based method [14]. Each of them has its own advantages and disadvantages, e.g., LRT is proven to be optimal, but it requires exact channel information and distributions of the primary signal and noise. The MF-based method needs perfect knowledge of the channel responses from primary users to the receiver and accurate synchronization (otherwise, its performance will dramatically be reduced) [15], it may not be possible if the primary users do not cooperate with the secondary users. The cyclostationary detection method requires the cyclic frequencies of the primary users, which may not be realistic for many spectrum reuse applications. Furthermore it needs high computation capabilities. The energy detection method does not require any primary signal information and it is robust to unknown dispersed channels and fading, but if the *SNR* of the received signal is low, the number of received signal samples is small and the power of noise is estimated inaccurately, the energy detection performance will decrease seriously [11]. The covariances-based method also does not require any prior information, but its computation complexity is also high [14].

As mentioned one drawback of the traditional energy detection is that if the *SNR* of received signal is low and the number of the received signal samples is small the corresponding performance may decrease seriously. In order to overcome this drawback a novel method with the purpose of obtaining a nonlinear threshold for energy detection based on PSO-SVM is proposed in this paper. The proposed method focuses on one single point and one antenna scenario, which can be divided into Offline module and Online module. In the Offline module, the proposed system generates two classes of training samples, one for the simulated situation that both signal and noise exist simultaneously while another case is for noise only. The normalized energy of these two classes of training samples is used as the classification feature to train the PSO-SVM. After each training step, a decision function is generated. In the Online module, the decision functions obtained in the Offline module are used as the nonlinear thresholds for energy detection to verify if the primary user is present. The experimental results show that the receiver operating characteristic (ROC) curve of proposed approach is much better than traditional energy detection. The rest of this paper is organized as follows: the PSO-SVM is introduced in Section 2. In Section 3 determination of threshold and theoretical analysis are proposed. Simulation results are given in Section 4. Conclusions are finally drawn in Section 5.

## 2. PSO-SVM

### 2.1. SVM

In this subsection, a brief introduction to SVM, proposed by Vapnik [16], is given. Let (**x***_i_*,*y_i_*)_1≤_*_i_*_≤_*_N_* be a set of *N* training samples, each sample **x***_i_* ∈ *R^d^* is a vector, *d* being the dimension of the input space, belonging to a class labeled by *y_i_* ∈ {1, −1}. It amounts to find weight vector **w** and scale *b*, which satisfy:

(1) $$y_{i}\left(\langle w\cdot x_{i}\rangle +b\right)\geq 1$$

where 〈·〉 is inner product. The aim of SVM is to find the hyper-plane which makes the samples with the same label on the same side of the hyper-plane. The quantity 1/‖*w*‖_2_ is the margin, and optimal separating hyper-plane (OSH) is the separating hyper-plane which maximizes the margin. The larger the margin, the better the generalization is expected [17]. To search the maximum *γ*, quadratic programming is usually used, leading to:

(2) $$\begin{array}{l} \underset{w,b}{\text{minimize}}\frac{1}{2}{\left\|w\right\|}^{2}\\ \text{subject}\;\text{to}\;y_{i}\left(\langle w\cdot x_{i}\rangle +b\right)\geq 1,i=1,2,\cdots ,N \end{array}\}$$

according to Equation (2), a hyper-plane 〈**w**·**x***_i_*〉 + *b* = 0 with the largest margin can be obtained. For equality constraints in Equation (2), they can be modified to unconstrained by integrating positive Lagrange multipliers, leading to:

(3) $$L\left(w,b,\alpha \right)=\frac{1}{2}\langle w\cdot w\rangle -\sum_{i=1}^{N}\alpha_{i}\;\left[y_{i}\left(\langle w\cdot x_{i}\rangle +b\right)-1\right]$$

to minimize *L*(**w**, *b*, *α*) by requiring the gradient of *L*(**w**, *b*, *α*) respect to **w** and *b* vanish, a dual form is given by:

(4) $$\begin{array}{l} w=\sum_{i=1}^{N}\alpha_{i}y_{i}x_{i}\\ \sum_{i=1}^{N}\alpha_{i}y_{i}=0\\ \alpha_{i}\geq 0 \end{array}$$

substituting Equation (4) into Equation (3) gives:

(5) $$\begin{array}{l} \;\underset{w,b}{\inf}L\left(w,b,\alpha \right)\\ =\frac{1}{2}\sum_{i=1}^{N}y_{i}y_{j}\alpha_{i}\alpha_{j}\;\langle x_{i}\cdot x_{j}\rangle -\sum_{i=1}^{l}y_{i}y_{j}\alpha_{i}\alpha_{j}\;\langle x_{i}\cdot x_{j}\rangle +\sum_{i=1}^{l}\alpha_{i}\\ =\sum_{i=1}^{l}\alpha_{i}-\frac{1}{2}\sum_{i=1}^{l}y_{i}y_{j}\alpha_{i}\alpha_{j}\langle x_{i}\cdot x_{j}\rangle =w\left(\alpha \right) \end{array}$$

According to Equation (3) and Equation (5), it is obvious that $w\left(\alpha \right)=\underset{w,b}{\inf}L\left(w,b,\alpha \right)\leq L\left(w,b,\alpha \right)$, thus the quadratic programming problem in Equation (3) can be converted to:

(6) $$\text{maximize}w\left(\alpha \right)=\sum_{i=1}^{N}\alpha_{i}-\frac{1}{2}\sum_{i=1}^{N}y_{i}y_{j}\alpha_{i}\alpha_{j}\;\langle x_{i}\cdot x_{j}\rangle$$

subject to:

(7) $$\begin{array}{l} \sum_{i=1}^{N}\alpha_{i}y_{i}=0\\ \alpha_{i}\geq 0\;i=1,\cdots ,N\\ \alpha_{i}\left[y_{i}\left(\langle w\cdot x\rangle +b\right)-1\right]=0 \end{array}$$

the third constraint condition in Equation (7) is the Karush-Kuhn-Tucker (KKT) condition [18]. There is a Lagrange multiplier *a_i_* for each training sample, the training samples for which *a_i_* > 0 are called “support vectors”, lying on one of the two hyper-planes: 〈**w**·**x**^+^〉 + *b* = +1; 〈**w**·**x**^−^〉 + *b* = −1 The training samples will only appear in the form of inner products between vectors.

In the nonlinear case, the approach adapted to noisy data is to make a soft margin. We introduce the slack variables *ξ_i_* ≥ 0, *i* = 1, 2, …, *N* so that:

(8) $$y_{i}\left(\langle w\cdot x_{i}\rangle +b\right)\geq 1-\xi_{i}$$

The generalized OSH is the solution of minimizing:

(9) $$\frac{1}{2}\langle w\cdot w\rangle +C\sum_{i=1}^{N}\xi_{i}$$

subject to Equation (7). The parameter $\sum_{i=1}^{N}\xi_{i}$ is the upper bound of the number of training errors and *C* is the penalty parameter to control errors.

In the nonlinear SVM, a kernel function is introduced to map the initial data into a feature space with a high dimension. In the new space, the data should be linearly separable. Then Equation (6) can be converted to:

(10) $$\text{maximize}\;w\left(\alpha \right)=\sum_{i=1}^{N}\alpha_{i}-\frac{1}{2}\sum_{i=1}^{N}y_{i}y_{j}\alpha_{i}\alpha_{j}K\;\left(x_{i},x_{j}\right)$$

subject to Equation (7), and 0 ≤ *α_i_* ≤ *C*. K(**x***_i_*, **x***_j_*) is the kernel function. As one of the most popular kernel functions, the RBF kernel function is considered in this paper, and it takes the following form:

(11) $$K\left(x_{i},x_{j}\right)=\exp\left\{-g{\left\|x_{j}-x_{i}\right\|}^{2}\right\}$$

where *g* is kernel parameter, and denoting the width of kernel function. If *g* is too big, the SVM may outfit the training data, and a too small *g* may make SVM algorithm not flexible enough for complex function approximation. By solving Equation (10) we can obtain the minimum 〈**w**·**w**〉:

(12) $$\langle w^{*}\cdot w^{*}\rangle =\sum_{i=1}^{N}y_{i}y_{j}{\alpha_{i}}^{*}{\alpha_{j}}^{*}K\left(x_{i},x_{j}\right)$$

where w*, $\alpha_{i}^{*}$ are the solution of Equation (10). Then the decision function is:

(13) $$f\left(x\right)=\text{sign}\left(\langle w^{*}\cdot x\rangle +b\right)$$

where **x** is a test sample with an unknown label *y*. Noting that w* only depend on the training samples (**x***_i_*, *y_i_*)_1≤_*_i_*_≤_*_N_*, if we choose a set of training samples (**x***_i_*, *y_i_*)_1≤_*_i_*_≤_*_N_*, a unique decision function will be obtained by solving Equation (10).

### 2.2. Particle Swarm Optimization

The parameters *C* and *g* need to be set to solve Equation (10). However, in a practical situation it is difficult to set these two parameters properly. In this subsection, we integrate a PSO (particle swarm optimization) method to adaptively set *C* and *g*.

Particle Swarm Optimization (PSO) is inspired by the social behavior of birds, birds in a swarm preying on food and cooperation with each other to search for the optimal position to obtain the food. In a PSO optimal problem, there are a group of individuals, each individual is called a “particle”, which may be a potential solution. Suppose that the solution space of optimization problem is *D* dimensions. The *i-th* particle is *X_i_* = (*x_i_*_1_, *x_i_*_2_, *x_i_*_3_, …*x_iD_*), the optimal position for itself is *P_i_* = (*p_i_*_1_, *p_i_*_2_, *p_i_*_3_, …*p_iD_*) and its speed move to this position is *V_i_* = (*v_i_*_1_, *v_i_*_2_, *v_i_*_3_, …*v_iD_*), the optimal swarm position is *P_g_* = (*p_g_*_1_, *p_g_*_2_, *p_g_*_3_, …*p_gD_*), iteration to find the optimal position is given by:

(14) $$x_{id}^{t+1}=\varpi \cdot v_{id}^{t}+c_{1}\cdot r_{1}\cdot \left(p_{id}-x_{id}^{t}\right)+c_{2}\cdot r_{2}\cdot \left(p_{gd}-x_{id}^{t}\right)$$

(15) $$x_{id}^{t+1}=x_{id}^{t}+v_{id}^{t+1}$$

where $x_{id}^{t}$ and $v_{id}^{t}$ are the *d*–*th* location and speed component of *i*−*th* particle at the *t*−*th* iteration, *c*_1_ and *c*_2_ are two positive coefficients, *r*_1_ and *r*_2_ are the random number selected from 0 to 1, *ϖ* is the flexible coefficient of $v_{id}^{t}$. The second part of Equation (12) is called “cognitive” part which is the optimization of the particle itself, and the third part of Equation (12) is the “swarm part”, which is the cooperation with other particles. In addition, a boundary for each particle should be set as, [−*x_id_*_max_, *x_id_*_max_] and *v_id_*_max_. In the process of the iteration, if the parameter of particle is out of the range, they are replaced by the boundary value. For the optimization of the error penalty parameter *C* and kernel parameter *g*, each particle is set as (*C*, *g*), and a three cross validation is used to estimate the performance of each (*C*, *g*), training data is separated into three parts, one part is considered as the validation set and the remaining two parts are for training. Our earlier work has validated this PSO algorithm [19].

## 3. Threshold Determination and Theoretical Analysis

Common notation as summarized in Table 1 is used throughout this section.

### 3.1. Basic Conception of Energy Detection

In this subsection we introduce the general model for spectrum sensing, then review the energy detection scheme and analyze the relationship between the probability of false alarm and probability of detection.

Suppose that we are interested in the frequency band with carrier frequency *f_c_*, bandwidth *W* and the received signal is sampled at sampling frequency *f_s_*, respectively. When the primary user is active, the discrete received signal at the secondary user is given by [5]:

(16) z(n)=s(n)+u(n)

where *z*(*n*) is under hypothesis *H*_1_. When the primary user is inactive, the received signal is given by:

(17) z(n)=u(n)

and this case is referred to the hypothesis *H*_0_. In this paper we only focus on one single point and one antenna spectrum sensing scenario, thus for simplicity we make the following assumptions [5]:

(AS1) The primary signal *s*(*n*) is an independent, and identically distributed (IID) random process with mean zero and unknown variance $E\left[{\left|s(n)\right|}^{2}\right]=\sigma_{s}^{2}$;

(AS2) The noise *u*(*n*) is a Gaussian IID random process with mean zero and variance $E\left[{\left|u(n)\right|}^{2}\right]=\sigma_{u}^{2}$, which can be estimated;

(AS3) The primary signal *s*(*n*) is independent of the noise *u*(*n*).

The signal-to-noise ratio (*SNR*) of the actual primary user measured at the secondary receiver of interest is $\lambda =\frac{\sigma_{s}^{2}}{\sigma_{u}^{2}}$, under hypothesis *H*_1_. We consider the circularly symmetric complex Gaussian (CSCG) as the noise case. For the primary signal *s*(*n*), we consider complex PSK modulated signal.

Two probabilities are of interest for spectrum sensing: probability of detection *P_d_*, which defines, at hypothesis *H*_1_, the probability of sensing method correctly detecting the presence of primary user; and probability of false alarm *P_f_*, which defines, at hypothesis *H*_0_, the probability of sensing method claiming the presence of primary user.

Energy detection is one of the most popular spectrum sensing schemes, because of it does not need any prior information about the primary signal and is easy to apply. Let *τ* be the available sensing time and *N_s_* be the number of samples, for simplicity we assume *N_s_* = *τf_s_*. The test statistics are given by [5]:

(18) $$\begin{array}{l} T=\frac{1}{N_{s}}\sum_{n=1}^{N_{s}}{\left|z\left(n\right)\right|}^{2}\\ T_{1}=\frac{1}{N_{s}}\sum_{n=1}^{N_{s}}{\left|s\left(n\right)+u\left(n\right)\right|}^{2}\\ T_{0}=\frac{1}{N_{s}}\sum_{n=1}^{N_{s}}{\left|u\left(n\right)\right|}^{2} \end{array}$$

for generality, decision statistics need to be normalized with the estimated power of noise, then the normalized decision statistics are given by:

(19) $$\begin{array}{l} T^{'}=\frac{T}{\sigma_{u}^{2}}\\ T_{1}^{'}=\frac{T_{1}}{\sigma_{u}^{2}}\\ T_{0}^{'}=\frac{T_{0}}{\sigma_{u}^{2}} \end{array}$$

if the number of signal samples *N_s_* is small, the probability of false alarm *P_f_* for a predefined threshold *ε* is given by:

(20) $$P_{f}=\mathrm{Pr}\left(T^{'}>\varepsilon |H_{0}\right)=\mathrm{Pr}\left(T_{0}^{'}>\varepsilon \right)$$

The far right-hand side of Equation (20) indicates a class of chi-square variable with 2*N_s_* degrees of freedom for complex-valued case. From Equation (20) the threshold *ε* is related to the *P_f_* as *ε* = *chi*2^−1^(*P_f_*, 2*N_s_*), where *chi*2^−1^(·) is inverse of the chi-square cumulative distribution function. For the same threshold *ε*, the probability of detection *P_d_* is given by:

(21) $$P_{d}=\mathrm{Pr}\left(T^{'}>\varepsilon |H_{1}\right)=\mathrm{Pr}\left(T_{1}^{'}>\varepsilon \right)$$

The symbol $T_{1}^{'}$ indicates a class of non-central chi-square variable with 2*N_s_* degrees of freedom and a non-centrality parameter *λ*, in our case, $\lambda =\frac{\sigma_{s}^{2}}{\sigma_{u}^{2}}$, extensive tables exist for the chi-square distribution, but the non-central chi-square has not been as extensively tabulated.

In this paper, we use approximations proposed by Patnaik [20] to replace the non-central chi-square with a central chi-square having a different number of degrees of freedom and a modified threshold level, If the non-central chi-square variable has 2*N_s_* degrees of freedom and non-centrality parameter *λ*, define a modified number of degrees of freedom *D* and a threshold divisor *G* given by:

(22) $$\begin{array}{c} D={\left(2N_{s}+\lambda \right)}^{2}/\left(2N_{s}+2\lambda \right)\\ G=\left(2N_{s}+2\lambda \right)/\left(2N_{s}+\lambda \right) \end{array}$$

then:

(23) $$P_{d}=\mathrm{Pr}\left(T_{1}^{'}>\varepsilon \right)=\mathrm{Pr}\left(T_{0}^{'}>\varepsilon /G\right)$$

As mentioned above, when the probability of false alarm *P_f_* and the number of samples *N_s_* is set, we can obtain a unique value of threshold *ε*, it equals to a linear classifier in binary classification problem. But if the *SNR λ* is low while the number of signal samples *N_s_* is small, the corresponding spectrum sensing problem is linearly inseparable, and the traditional energy detection can not classify this linearly inseparable problem efficiently.

### 3.2. Nonlinear Threshold System

**Figure 1.**
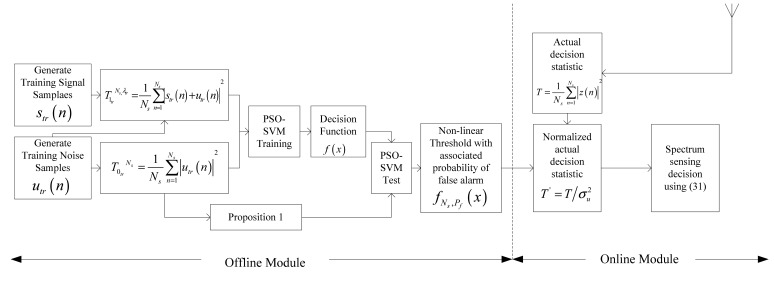
System model of the proposed method.

To overcome the drawbacks of traditional energy detection mentioned above, the authors here propose a method in purpose of obtaining nonlinearly threshold based on PSO-SVM. The system process encompasses two distinct modules *i.e.*, Offline and Online, which are clearly illustrated by the Figure 1.

#### 3.2.1. Offline Module

The main function of the Offline Module is to generate the nonlinear thresholds for energy detection.

Firstly, the proposed system generates training signal and training noise under AS1-3. Therefore the variance of training signal is a known value $\sigma_{s_{tr}}^{2}=\lambda_{tr}$, the variance of training noise is $\sigma_{u_{tr}}^{2}=1$, and training *SNR* is *λ_tr_*.

Secondly, based on the parameter *λ_tr_* and number of signal samples *N_s_*, two classes of training decision statistics under hypothesis *H*_0_ and hypothesis *H*_1_ could be obtained, which are given by:

(24) $${T_{1_{tr}}}^{N_{s,}\lambda_{tr}}=\frac{1}{N_{s}}{\sum_{n=1}^{N_{s}}\left|s_{tr}\left(n\right)+u_{tr}\left(n\right)\right|}^{2}$$

(25) $${T_{0_{tr}}}^{N_{s}}=\frac{1}{N_{s}}{\sum_{n=1}^{N_{s}}\left|u_{tr}\left(n\right)\right|}^{2}$$

where the symbol $T_{1_{tr}}^{N_{s},\lambda_{tr}}$ indicates a class of non-central chi-square variables with mean 1+*λ_tr_* 2*N_s_* degrees of freedom, and a non-centrality parameter *λ_tr_*. While the symbol ${T_{0_{tr}}}^{N_{s}}$ indicates a class of central chi-square variables with mean 1 and 2*N_s_* degrees of freedom.

Thirdly, labeling each variable of $T_{1_{tr}}^{N_{s},\lambda_{tr}}$ class as “+1”, and each variable of ${T_{0_{tr}}}^{N_{s}}$ class as “−1”, then these two classes of variables are used as training data to train PSO-SVM mentioned in Section 2. Consequently, a separating hyper-plane 〈w*·**x** 〉 + *b* = 0 and a decision function *f*(*x*) = *sign*(〈w*·**x** 〉 + *b*)could be derived.

In the fourth step, the variables of ${T_{0_{tr}}}^{N_{s}}$ class are applied to test this decision function so as to gain the probability (denoted as $P_{e}^{N_{s},\lambda_{tr}}$) that the decision function mistakenly label a ${T_{0_{tr}}}^{N_{s}}$ variable as a $T_{1_{tr}}^{N_{s},\lambda_{tr}}$ variable.

##### Proposition1

The probability of a variable mistakenly labeled by decision function is determined by the geometric distance *γ* which is from this variable to the separating hyper-plane.

##### Proof

The proof is mainly based on Rosenblatt classifier, and detailed proof is given in Appendix A.

The average geometric distance from variables of ${T_{0_{tr}}}^{N_{s}}$ class to separating hyper-plane is given by:

(26) $$\gamma_{-1_{tr}}\;=E\left[\frac{\left|\langle w^{*}\cdot T_{0_{tr}}^{N_{s}}\rangle +b\right|}{{\left\|w^{*}\right\|}_{2}}\right]=\frac{\left|\langle w^{*}\cdot 1\rangle +b\right|}{{\left\|w^{*}\right\|}_{2}}$$

while the average geometric distance from variables of $T_{0}^{\prime }$ class to separating hyper-plane is given by:

(27) $$\gamma_{-1}\;=E\left[\frac{\left|\langle w^{*}\cdot T_{0}^{\prime }\rangle +b\right|}{{\left\|w^{*}\right\|}_{2}}\right]=\frac{\left|\langle w^{*}\cdot 1\rangle +b\right|}{{\left\|w^{*}\right\|}_{2}}$$

according to Equation (26) and Equation (27), it is obvious that the average geometric distance from variables of ${T_{0_{tr}}}^{N_{s}}$ class to the separating hyper-plane is equal to the average geometric distance from variables of $T_{0}^{\prime }$ class to the separating hyper-plane, and the probability distribution of ${T_{0_{tr}}}^{N_{s}}$ class are same as the probability distribution of $T_{0}^{\prime }$ class. Then, based on proposition 1, the probability that a variable of ${T_{0_{tr}}}^{N_{s}}$ class mistakenly labeled as $T_{1_{tr}}^{N_{s},\lambda_{tr}}$ class is equal to the probability (the probability of false alarm) that a variable of $T_{0}^{\prime }$ class is mistakenly labeled as $T_{1}^{\prime }$ class. To conclude, it could be expressed as equation:

(28) $$P_{e}^{N_{s},\lambda_{tr}}=\mathrm{Pr}(f\left({T_{0_{tr}}}^{N_{s}}\right)=1)=\mathrm{Pr}(f\left({T_{0}}^{\prime }\right)=1)=P_{f}$$

Each set of parameter *λ_tr_* and *N_s_* will be used to generate two classes of variable: $T_{1_{tr}}^{N_{s},\lambda_{tr}}$ and ${T_{0_{tr}}}^{N_{s}}$. A decision function marked as *f_N_s_, P_f__*(*x*) could be obtained by training PSO-SVM with these two classes of data. Finally, the decision function *f_N_s_, P_f__*(*x*) is stored as a non-linear threshold.

**Figure 2.**
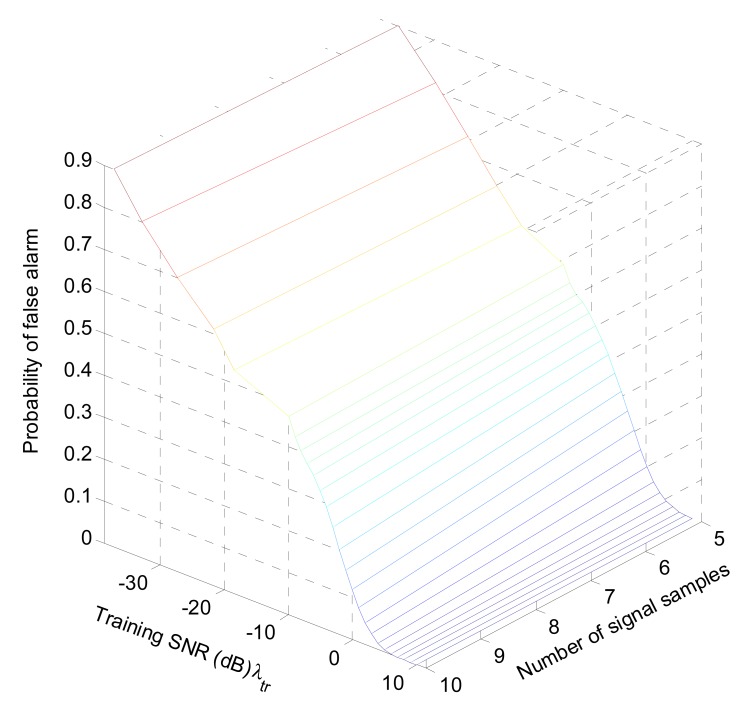
Training Results of Table 3.

The process to obtain a non-linear threshold is shown in Table 2, and some of typical training results of *f_N_s_, P_f__*(*x*) are shown in Table 3 and Figure 2.

#### 3.2.2. Online Module

In the Online module, the proposed system automatically chooses one of the decision functions (according to required number of signal samples *N_s_* and probability of false alarm *P_f_*) stored in the Offline Module as non-linear threshold to judge whether the actual primary user is present e.g., the required number of signal samples is *N_s_* = 5 and probability of false alarm is *P_f_* = 0.1, the proposed system would apply decision function *f*_5,0.1_(*x*) as the non-linear threshold.

If a decision function *f_N_s_, P_f__*(*x*) is chosen as the nonlinear threshold, Equations (20) and (21) will be converted to:

(29) $$P_{f}=\mathrm{Pr}(f_{N_{s},P_{f}}\left(T^{'}\right)=1|H_{0})=P_{e}^{N_{s},\lambda_{tr}}$$

(30) $$P_{d}=\mathrm{Pr}(f_{N_{s},P_{f}}\left(T^{'}\right)=1|H_{1})$$

The result of spectrum sensing is given by:

(31) $$\left\{ \begin{array}{l} f_{N_{s},P_{f}}\left(T^{'}\right)=1,\;\text{Primary user is presented}\;\left(\text{hypothesis}\;H_{1}\right)\\ f_{N_{s},P_{f}}\left(T^{'}\right)=-1,\;\text{Primary user is not presented}\;\left(\text{hypothesis}\;H_{0}\right) \end{array}\right.$$

#### 3.2.3. Comparison with Traditional Energy Detection

Energy detection is the basic sensing method, which was first proposed in [9] and further studied in [5,10]. It does not need any information of the signal to be detected and is robust to unknown dispersive channels. Energy detection compares the normalized average power of the actual received signal plus noise $T_{1}^{\prime }=T_{1}/\sigma_{u}^{2}$ variable with the noise power ${T_{0}}^{'}=T_{0}/\sigma_{u}^{2}$ to make a decision. To guarantee a reliable detection, the threshold must be set according to the actual noise power $\sigma_{u}^{2}$ and the number of samples *N_s_* [9]. The difference between the traditional Energy detection and the proposed system is that the proposed system has a Offline module to obtain decision functions as the non-linear thresholds.

In the Offline module, the system needs a great number of $T_{1_{tr}}^{N_{s},\lambda_{tr}}$ variables and ${T_{0_{tr}}}^{N_{s}}$ variables to train PSO-SVM during each training process. Taking the simulation process in this article as an instance, 500 variables from $T_{1_{tr}}^{N_{s},\lambda_{tr}}$ and the same from ${T_{0_{tr}}}^{N_{s}}$ are deployed for each training process, of which computational complexity is about *O*(1000^3^) [16].

As the price of getting the full list of decision functions, the training times are huge. Therefore the overall computational complexity of the offline module is extremely high. However, in a real spectrum sensing situation, we only take care of the computational complexity in the Online module. The computational complexity of traditional Energy detection needs about *N_s_* multiplications and additions. Hence, the computational complexity of the proposed methods is about *N_s_* + 1 multiplications and additions, which is competitive with traditional Energy Detection.

## 4. Results and Discussion

In this section, we use the decision functions stored in the Offline module as the nonlinear thresholds to simulate the probability of detection *P_d_* in the Online module.

**Figure 3.**
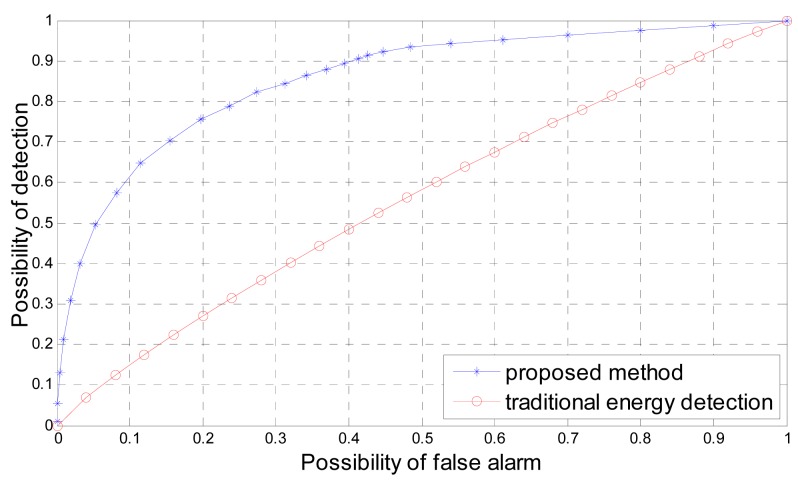
Receiver operating characteristic curve of the proposed method and traditional energy detection for number of signal samples *N_s_* = 5 and actual *SNR λ* = 0 *dB*.

In Figure 3 we compare the receiver operating characteristic curve of the proposed method and traditional energy detection for actual *SNR λ* = 0*dB* and number of signal samples *N_s_* = 5. If the *SNR* of the actual received signal is low and the number of signal samples is small e.g., *λ* = 0 *dB* and *N_s_* = 5, the corresponding spectrum sensing problem based on energy detection is a linearly inseparable binary classification problem. Traditional energy detection with a predefined threshold is a linear classifier, it cannot solve linearly inseparable problem efficiently. But the proposed method is a nonlinear classifier based on PSO-SVM, which can solve linearly inseparable problem efficiently. As shown in Figure 3, the performance of the proposed method is much better than traditional energy detection.

**Figure 4.**
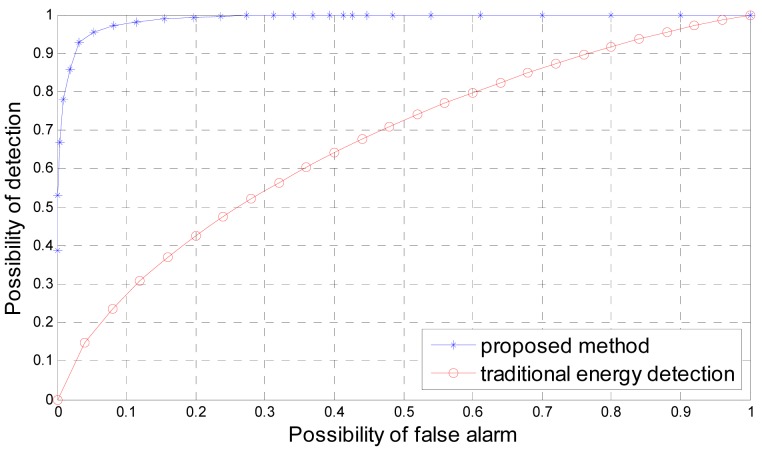
Receiver operating characteristic curve of the proposed method and traditional energy detection for *P_d_* for number of signal samples *N_s_* = 5 and actual *SNR λ* = 5 *dB*.

In Figure 4 we compare the receiver operating characteristic curve of the proposed method and traditional energy detection in terms of actual *SNR λ* = 5 *dB* and number of signal samples *N_s_* = 5. Although the actual *SNR λ* increases to 5 *dB* but the number of signal samples *N_s_* = 5 is small, which means the corresponding spectrum sensing problem based on energy detection is still linearly inseparable. Therefore, as shown in Figure 4, the performance of proposed method is dramatically better than the traditional energy detection method.

**Figure 5.**
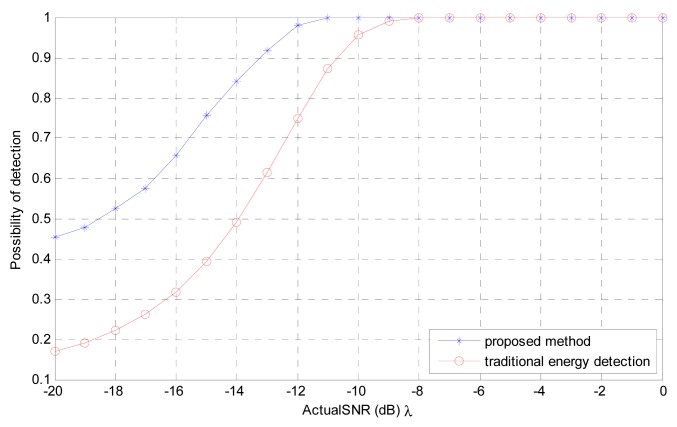
*P_d_* of the proposed method and traditional energy detection for number of signal samples, *N_s_* = 1,000, *P_f_* = 0.1 and actual *SNR λ* = −20 *dB* −0 *dB*.

In Figure 5 we compare *P_d_* of the proposed method and traditional energy detection for fixed *P_f_* = 0.1 and number of signal samples *N_s_* = 1,000, the corresponding performance of the proposed method is still better than energy detection. This is because although the number of sensing samples is large *i.e.*, 1000, but actual *SNR λ* is low, thus the corresponding spectrum sensing problem is linearly inseparable, however the proposed method can classify linearly inseparable problem efficiently. As shown in Figure 5, at *λ* = −20 *dB* the proposed method is almost three times better than traditional energy detection. With the *λ* increases the spectrum sensing problem coverage to linearly separable, and the difference of performance between the proposed method and the traditional energy detection also decreases.

## 5. Conclusions

In this paper, a novel modular spectrum sensing method for cognitive radio based on PSO-SVM is proposed. It comprises two distinct modules, *i.e.*, Offline and Online. In the Offline module, the decision functions with associated probabilities of false alarm are obtained. In the Online module, the primary user is detected by using the decision functions obtained in the Offline module.

The proposed method actually is independent from the traditional detection method, a nonlinear decision is exploited to replace the linear threshold, which drastically improves the performance of detection without increasing the computational complexity in Online phase. The approach can be used for various signal detection applications without *a priori* knowledge of signals and channels. Simulations have been carried to evaluate the performance of the proposed method. It has been shown that the proposed approach is more effective than the traditional energy detection approach in hostile environments. More specifically, when the received signal samples are lacking and *SNR* is low, the approach proposed in this paper can give a reliable performance, while the traditional energy detection approach is hypodynamic.

In our future research work, we will try to apply the proposed method to enhance more sophisticated detection algorithm which uses predefined linear threshold (e.g., the method proposed in [14]). More specifically, secondary users are located with detected radio map thereby deploying space-time spectrum sensing. And the specific signal pattern from the primary user can be recognized by analyzing the signal detected.

## Figures and Tables

**Table 1.** Notation

*N_s_*	**number of signal samples for spectrum sensing**
*s* (*n*)	actual received primary signal sample
*u* (*n*)	actual received noise sample
*s_tr_* (*n*)	training primary signal sample in offline module
*u_tr_* (*n*)	training noise sample in offline module
$\sigma_{s}^{2}$	variance of actual received primary signal
$\sigma_{u}^{2}$	variance of actual noise
$\sigma_{s_{tr}}^{2}=\lambda_{tr}$	variance of training primary signal in offline module
$\sigma_{u_{tr}}^{2}=1$	variance of training noise in offline module
*λ*	*SNR* of actual received primary signal
*λ_tr_*	*SNR* of training primary signal in offline module
*T*	actual decision statistic
*T*_1_	actual decision statistic at hypothesis *H*_1_
*T*_0_	actual decision statistic at hypothesis *H*_0_
$T^{'}=T/\sigma_{u}^{2}$	normalized actual decision statistic
$T_{1}^{\prime }=T_{1}/\sigma_{u}^{2}$	normalized actual decision statistic at hypothesis *H*_1_
$T_{0}^{\prime }=T_{0}/\sigma_{u}^{2}$	normalized actual decision statistic at hypothesis *H*_0_
$T_{1_{tr}}^{N_{s},\lambda_{tr}}$	training decision statistic defined by *N_s_*, *λ_tr_* at hypothesis *H*_1_ in offline module
${T_{0_{tr}}}^{N_{s}}$	training decision statistic defined by *N_s_* at hypothesis *H*_0_ in offline module

**Table 2.** The process to obtain a non-linear threshold.

1. Generate training signal *s_tr_* (*n*) and training noise *u_tr_* (*n*), with $\sigma_{s_{tr}}^{2}=\lambda_{tr}$, $\sigma_{s_{tr}}^{2}=1$.
2. Compute two classes of data: $T_{1_{tr}}^{N_{s},\lambda_{tr}}$, ${T_{0_{tr}}}^{N_{s}}$ by (24) and (25).
3. Train PSO-SVM with two classes of data: $T_{1_{tr}}^{N_{s},\lambda_{tr}}$, ${T_{0_{tr}}}^{N_{s}}$ to obtain a decision function *f* (*x*).
4. Test this *f* (*x*) with the variables of ${T_{0_{tr}}}^{N_{s}}$ class to obtain $P_{e}^{N_{s},\lambda_{tr}}$, based on Proposition 1 $P_{e}^{N_{s},\lambda_{tr}}=P_{f}$.
5: Return *f* (*x*) as *f_N_s_, P_f__*, and store it as non-linear threshold.

**Table 3.** Typical Training Results.

*N_s_* = 5			*N_s_* = 10		
*λ_tr_*	*P_f_*	*f_N_s_, P_f__*(*x*)	*λ_tr_*	*P_f_*	*f_N_s_, P_f__*(*x*)
−35.7 dB	0.9	*f*_5,0.9_(*x*)	−37.1 dB	0.9	*f*_10,0.9_(*x*)
−29.8 dB	0.8	*f*_5,0.8_(*x*)	−32.8 dB	0.8	*f*_10,0.8_(*x*)
−24.9 dB	0.7	*f*_5,0.7_(*x*)	−27.2 dB	0.7	*f*_10,0.7_(*x*)
−20.4 dB	0.6	*f*_5,0.6_(*x*)	−21.6 dB	0.6	*f*_10,0.6_(*x*)
−16.5 dB	0.5	*f*_5,0.5_(*x*)	−18.4 dB	0.5	*f*_10,0.5_(*x*)
−6.5 dB	0.4	*f*_5,0.4_(*x*)	−8.1 dB	0.4	*f*_10,0.4_(*x*)
−2.8 dB	0.3	*f*_5,0.3_(*x*)	−4.2 dB	0.3	*f*_10,0.3_(*x*)
−0.2 dB	0.2	*f*_5,0.2_(*x*)	−1.7 dB	0.2	*f*_10,0.2_(*x*)
2.3 dB	0.1	*f*_5,0.1_(*x*)	−0.2 dB	0.1	*f*_10,0.1_(*x*)

## Appendix: Proof of Proposition 1

If the point (**x**, *y*) is correctly classified by the optimal hyper-plane $y\frac{\left|\langle w\cdot x\rangle +b\right|}{{\left\|w\right\|}_{2}}\geq \gamma$. In order to facilitate the analysis, we introduce an additional coordinate to expand the sample, the new sample is **x̂** = (**x**′, *R*)′, *R* is a constant, **x**′ is the transpose of **x**. Similarly we can use bias *b* to expand the weighted vector *w*, and the expanded *w* is **ŵ** = (**w**′, *b*/*R*)′, the iteration starts from **ŵ** = 0, if there is a wrong separation, the **ŵ** is updated accordingly. Suppose **ŵ***_t_*_−1_ is the weighed vector before the wrong separation:

(32) $$\langle {\overset{\wedge }{w}}_{t-1}\cdot {\overset{\wedge }{x}}_{i}\rangle =y_{i}\left({\overset{}{w}}_{t-1}\cdot x_{i}+b_{t-1}\right)\leq 0$$

where (**x**, *y*) is the data mistakenly separated by ${\overset{\wedge }{w}}_{t-1}={\left(w_{t-1}^{'},b_{t-1}/R\right)}^{'}$ and the *t* − *th* update is given by:

(33) $${\overset{\wedge }{w}}_{t}={\left(w_{t}^{'},b_{t}/R\right)}^{'}={\left(w_{t-1}^{'},b_{t-1}/R\right)}^{'}+\alpha_{i}y_{i}{\left(x_{i}^{'},R\right)}^{'}={\overset{\wedge }{w}}_{t-1}+\alpha_{i}y_{i}{\overset{\wedge }{x}}_{i}$$

where *b_t_*/*R* = *b_t_*_−1_/*R* + *αyR*, *α* is Lagrange multiplier and we can obtain a result:

(34) $$\langle {\overset{\wedge }{w}}_{t}\cdot \overset{\wedge }{w}\rangle =\langle {\overset{\wedge }{w}}_{t-1}\cdot \overset{\wedge }{w}\rangle +\alpha y_{i}\langle {\overset{\wedge }{x}}_{i}\cdot \overset{\wedge }{w}\rangle \geq \langle {\overset{\wedge }{w}}_{t-1}\cdot \overset{\wedge }{w}\rangle +\alpha \gamma$$

Before the *t-th* mistaken separation there already has *t* − 1 wrong separations thus we can obtain:

(35) $$\langle {\overset{\wedge }{w}}_{t}\cdot \overset{\wedge }{w}\rangle \geq t\alpha \gamma$$

similarly:

(36) $$\begin{array}{ll} {\left\|\overset{\wedge }{w_{t}}\right\|}^{2} & ={\left\|{\overset{\wedge }{w}}_{t-1}\right\|}^{2}+2\alpha y\left(\overset{\wedge }{w_{t-1}}\cdot \overset{\wedge }{x}\right)+\alpha^{2}{\left\|\overset{\wedge }{x}\right\|}^{2}\\ & \leq {\left\|{\overset{\wedge }{w}}_{t-1}\right\|}^{2}+\alpha^{2}{\left\|\overset{\wedge }{x}\right\|}^{2}\leq {\left\|{\overset{\wedge }{w}}_{t-1}\right\|}^{2}+\alpha^{2}\left({\left\|\overset{\wedge }{x}\right\|}^{2}+R^{2}\right)\\ & \leq {\left\|{\overset{\wedge }{w}}_{t-1}\right\|}^{2}+2\alpha^{2}R^{2} \end{array}$$

which means:

(37) $${\left\|\overset{\wedge }{w_{t}}\right\|}^{2}\leq 2t\alpha^{2}R^{2}$$

and a close form is given by Equation (34) and Equation (35):

(38) $$\left\|\overset{\wedge }{w}\right\|\sqrt{2t}\alpha R\geq \left\|\overset{\wedge }{w}\right\|\left\|\overset{\wedge }{w_{t}}\right\|\geq \langle \overset{\wedge }{w_{t}}\cdot \overset{\wedge }{w}\rangle \geq t\alpha \gamma$$

based on Equation (35) and Equation (36):

(39) $$t\propto \frac{1}{\gamma }$$

Proposition 1 is proven.

## References

[1] (2002). Spectrum Policy Task Force Report; Technical Report TR 02-155.

[2] Mitola J., Maguire G.Q. (1999). Cognitive radio: Making software radios more personal. IEEE Personal Commun..

[3] Mitola J. (2000). Cognitive Radio: An Integrated Agent Architecture for Software Defined Radio. Ph.D. Thesis.

[4] Haykin S. (2005). Cognitive radio: Brain-Empowered wireless communications. IEEE J. Sel. Area Commun..

[5] Liang Y.C., Zeng Y.H., Peh E.C.Y., Hoang A.T. (2008). Sensing-throughput tradeoff for cognitive radio networks. IEEE Trans. Wirel. Commun..

[6] Kay S. (1998). Fundamentals of Statistical Signal Processing: Detection Theory.

[7] Quan Z., Cui S., Sayed A.H. (2008). Optimal linear cooperation for spectrum sensing in cognitive radio networks. IEEE J. Sel. Top. Signal Process..

[8] Taricco G. (2011). Optimization of linear cooperative spectrum sensing for cognitive radio networks. IEEE J. Sel. Top. Signal Process..

[9] Urkowitz H. (1967). Energy detection of unknown deterministic signals. Proc. IEEE.

[10] Sonnenschein A., Fishman P.M. (1992). Radiometric detection of spread-spectrum signals in noise of uncertainty power. IEEE Trans. Aerosp. Electron. Syst..

[11] Sahai A., Cabric D. Spectrum Sensing: Fundamental Limits and Practical Challenges.

[12] Gardner W.A. (1991). Exploitation of spectral redundancy in cyclostationary signals. IEEE Signal Process. Mag..

[13] Gardner W.A., Brown W.A., Chen C.K. (1987). Spectral correlation of modulated signals—Part II: Digital modulation. IEEE Trans. Commun..

[14] Zeng Y.H., Liang Y.C. (2009). Spectrum—Sensing algorithms for cognitive radio based on statistical covariances. IEEE Trans. Veh. Technol..

[15] Chen H.S., Gao W., Daut D.G. Signature Based Spectrum Sensing Algorithms for IEEE 802.22 WRAN.

[16] Vapnik V.N. (1995). The Nature of Statistical Learning Theory.

[17] Chapelle O., Haffner P., Vapnik V. (1999). Support vector machines for histogram-based image classification. IEEE Trans. Neural Netw..

[18] Kuhn H., Tucker A. Nonlinear Programming.

[19] Cai Z.R., Zhao H.L., Jia M. (2011). Spectrum sensing in cognitive radio based on adaptive optimal SVM. Inform. Technol. J..

[20] Patnaik P.B. (1949). The noncentral *χ*_2_ and *F* distributions and their applications. Biometrika.

